# Gene Expression Analysis and Validation of a Novel Biomarker Signature for Early-Stage Lung Adenocarcinoma

**DOI:** 10.3390/biom15060803

**Published:** 2025-05-31

**Authors:** Sanjan S. Sarang, Catherine M. Cahill, Jack T. Rogers

**Affiliations:** Neurochemistry Laboratory, Massachusetts General Hospital, Harvard Medical School, Boston, MA 02129, USA; ccahill@mgh.harvard.edu (C.M.C.); jackrogers@mgh.harvard.edu (J.T.R.)

**Keywords:** lung adenocarcinoma, differential gene expression, multiomics, protein–protein interactions, early-stage markers

## Abstract

Lung cancer is responsible for 2.21 million annual cancer cases and is the leading worldwide cause of cancer-related deaths. Specifically, lung adenocarcinoma (LUAD) is the most prevalent lung cancer subtype resulting from genetic causes; LUAD has a 15% patient survival rate due to it commonly being detected in its advanced stages. This study aimed to identify a novel biomarker signature of early-stage LUAD utilizing gene expression analysis of human lung tissue samples. Using 22 pairs of LUAD and matched normal lung microarrays, 229 differentially expressed genes were identified. These genes were networked for their protein–protein interactions, and 44 hub genes were determined from protein essentiality. Survival analysis of 478 LUAD patient samples identified four statistically significant candidates. These candidate genes’ expression profiles were validated from GTEx and TCGA (347 normal, 483 LUAD samples); immunohistochemistry validated the subsequent protein presence. Through intensive bioinformatic identification and multiple validations of the four-biomarker gene signature, AGER, MGP, and PECAM1 were identified as downregulated in LUAD; SLC2A1 was identified as upregulated in LUAD. These four biologically significant genes are involved in tumorigenesis and poor LUAD prognosis, meriting their use as a clinical biomarker signature and therapeutic targets for early-stage LUAD.

## 1. Introduction

Cancers are a modern threat to society and are classified by the uncontrolled growth and spread of abnormal cells throughout the body that can result in death, either with or without treatment [1]. Lung cancer, responsible for 2.21 million annual cases, is the leading worldwide cause of cancer-related deaths, with prognosis tightly linked to stage at diagnosis [2,3]. Lung cancers can be classified into two main subgroups: non-small-cell lung cancer (NSCLC), responsible for 85% of all lung cancer cases, and small-cell lung cancer, responsible for 15% of all lung cancer cases [2]. Specifically, lung adenocarcinoma (LUAD) is responsible for ten percent of all NSCLC cases in patients with no smoking history, suggesting that the incidence of LUAD is related to genetic variations and susceptibility [4]. In normal lung cells, proto-oncogenes are regulators of cell growth. However, modifications can occur in these genes, forming oncogenes and leading to carcinogenesis. These oncogenes can form through changes such as chromosomal translocation (e.g., chromosomal translocation of the *MYC* oncogene in Burkitt’s lymphoma), point mutation (e.g., point mutation at codon 12 of the RAS oncogene), and gene amplification (e.g., amplification of *c-MYC* in neuroblastoma) [5]. A second pivotal gene type that is involved in the pathogenesis of cancer is tumor suppressor genes. In normal cells, these genes regulate cell growth and block cancer; mutations that reduce the presence or activation of these genes lead to cancer. For example, *P53* is a tumor suppressor gene that is a tetramer and, when mutated, can lead to significant effects as the tetramer will always contain at least one mutant *P53* protein [5]. In LUAD, genetic abnormalities occur in normal alveolar and bronchial epithelial cells, promoting precancerous lesions such as atypical adenomatous hyperplasia (AAH). Epidermal growth factor receptor or anaplastic lymphoma kinase mutations within these lesions lead to elevated cell proliferation that allows these cells to survive and accumulate mutations [6]. Further mutations then cause these AAH lesions to progress into adenocarcinoma in situ (AIS), a non-invasive stage characterized by cellular atypia and architectural distortion [7]. With the AIS lesions acquiring more genetic instability, the lesions become invasive adenocarcinoma, penetrating the basement membrane and spreading to surrounding tissues [8].

The identification of markers in LUAD is critical as it can allow for diagnosing LUAD in its earlier stages and may enable timely intervention [9]. Low-dose CT (LDCT) screening can improve early detection and reduce mortality by approximately 20% in high-risk smokers. However, it suffers from frequent false positives and overdiagnosis [3,10]. These issues underscore a specific need for biomarkers to refine the screening and diagnosis of LUAD. Furthermore, utilizing a biomarker signature, a composite of multiple biomarkers can enhance diagnostic accuracy by integrating numerous molecular insights and minimizing variability [11]. Previous biomarker identification studies have been performed using bioinformatic analysis of gene data or laboratory tests and have discovered many biomarkers for various diseases [12,13,14,15,16]. Despite this, very few clinically recognized biomarkers for early-stage LUAD [12,13] are currently available. Identifying biomarkers for early-stage LUAD diagnosis is essential, as LUAD is a prominent cancer usually detected in its advanced stages [13,17]. At these advanced stages, there is a 15% survival rate. Early detection of LUAD through an accurate biomarker could allow for treatments to be conducted on a less advanced tumor, leading to an improved patient outcome [12,13,17].

This research aimed to identify a predictive biomarker signature of unique cancer-related genes that can be utilized for the early-stage diagnosis of LUAD to reduce disease mortality. In this investigation, we identified a novel biomarker signature to aid in predicting poor LUAD prognosis via early-stage samples.

Currently, many clinically recognized biomarkers of LUAD, such as carcinoembryonic antigen and CYFRA21-1, have limited sensitivity and specificity considering an application in early-stage disease [18]. Our research identified a biomarker signature comprising *AGER*, *MGP*, *PECAM1*, and *SLC2A1*, which uniquely integrates various relevant pathways such as cell adhesion, angiogenesis, and metabolic reprogramming, which improves the overall specificity and diagnostic confidence as opposed to single-marker approaches [19]. The signature utilizes the novel markers *AGER* and *MGP*; the downregulation of these markers has rarely been explored in LUAD [20,21]. In summary, the utilization of a biomarker signature with relevant markers increases diagnostic accuracy beyond single-marker approaches [10].

## 2. Materials and Methods

### 2.1. Data Acquisition

The transcriptomics dataset GSE32863 was obtained from the Gene Expression Omnibus (GEO), and the dataset was analyzed using the GEO2R tool. The dataset comprises gene expression profiles from 58 primary LUAD tumor tissues and 58 matched adjacent normal lung tissues [13]. The analysis was focused on analyzing 22 tumors classified as stages I-II and the 22 associated matched normal samples. All microarray data were derived from a high-density oligonucleotide platform that covers approximately 21,000 genes [13]. Each sample was assigned labels via the define feature, and the grouping was verified before proceeding with the analysis.

### 2.2. Differential Gene Expression Analysis

To accurately identify differentially expressed genes (DEGs), the Benjamini–Hochberg adjustment method was applied to the GEO2R analysis tool. Additionally, a highly stringent adjusted *p*-value (adj. *p*) cutoff of adj. *p* ≤ 0.01 was utilized to ensure statistical significance. Furthermore, a Log_2_ Fold Change (Log_2_FC) cutoff of | Log_2_FC | ≥ 2 was used to determine up- and downregulated DEGs [22]. Utilizing R version 4.3.2 (R Foundation for Statistical Computing, Vienna, Austria) data quality and normalization were verified by evaluating median-centered expression distributions for all selected samples in numerous comparative boxplots. The combination of cutoffs and validation of median-centered ensured that only the most significant and biologically relevant changes were selected for further downstream analysis.

### 2.3. Protein–Protein Interaction Networks and Hub Gene Identification

Once all genes were categorized as upregulated or downregulated, the entire set was uploaded into the Search Tool for the Retrieval of Interacting Genes/Proteins (STRING v11.5; https://string-db.org, ELIXIR Core Data Resource, EMBL, Heidelberg, Germany) to construct a protein–protein interaction (PPI) network. The network was then exported into Cytoscape v3.26.0 (The Cytoscape Consortium, San Diego, CA, USA; https://cytoscape.org), where the cytoHubba plugin v0.1 (developed by Institute of Systems Biology, National Tsing Hua University, Hsinchu, Taiwan) was used to identify hub genes. Using the two most robust centrality algorithms, Maximal Clique Centrality (MCC) and Density of Maximum Neighborhood Component (DMNC), both algorithms computed the shortest path to identify hub genes. In constructing the protein–protein interaction network, interactions with a confidence score of ≥0.4 were considered significant participants in the network [23]. Using the two topological algorithms, MCC and DMNC, nodes were ranked by centrality; a combination of both computations was used to determine the final hub gene set for further analysis. This approach considered overlapping high-centrality nodes from both methods, which elevated the confidence that the hub genes were pivotal in the LUAD network.

### 2.4. Kaplan–Meier Survival Analysis

Hub genes were exported into the Gene Expression Profiling Interactive Analysis 2 (GEPIA-2, v2.0; http://gepia2.cancer-pku.cn, Peking University, Beijing, China) platform to generate Kaplan–Meier survival curves, which visualize the correlation between gene expression levels with the overall survival (OS) of LUAD patients. The LUAD patient data were imported from The Cancer Genome Atlas (TCGA) and Genotype-Tissue Expression (GTEx) database. Genes that were observed to have significant survival correlation were retained, thus highlighting their prognostic potential in earlier-stage LUAD. Genes with log-rank *p*-values < 0.05 in the Kaplan–Meier analysis were deemed to be associated with overall survival. The default median expression cutoff in GEPIA-2 allowed for the high and low expression groups to be dichotomized [24]. The genes that met the criterion were retained as candidates for prognostic potential, allowing the subsequent analysis to focus on the most robust survival correlations.

### 2.5. Transcriptomic and Proteomic Validation

After OS analysis, the smaller set of key genes were validated for their overall differential expression using the “Expression DIY” tool in GEPIA-2, which accessed TCGA and GTEx for LUAD and healthy tissue expression data. Due to the high specificity of antigen–antibody binding reactions, immunohistochemistry (IHC) stains from the Human Protein Atlas (HPA) (https://www.proteinatlas.org/, Science for Life Laboratory, Uppsala University, Uppsala, Sweden) were used to validate the presence of proteins encoded by the biomarker genes. For the immunohistochemical validation, lung tissue staining was retrieved for each available candidate gene from the HPA [25]. The HPA provided results from three independent normal lung tissue subjects and five to six independent LUAD tumor tissue subjects for each gene, enabling a comparison between protein presence in normal and cancerous tissues. The staining level and intensity strength were reported.

## 3. Results

### 3.1. Identification of DEGs

**Figure 1:**
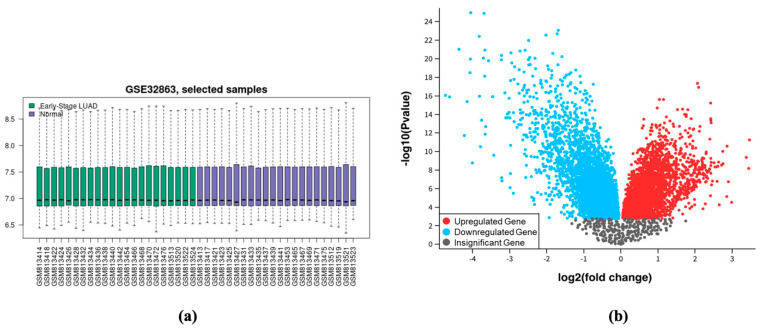
Boxplot of sample expression and volcano plot of DEGs from GSE32863. (**a**) Boxplot of selected samples: median centering shows the statistical correction to be accurate and the samples ready for further analysis. (**b**) Volcano plot of DEGs: each dot represents a gene regulation and significance prediction.

The GSE32863 expression profile was downloaded from GEO and included LUAD tumor and normal lung samples for 22 unique patients, which assayed 21,044 distinct genes per sample as described by Selamat et al. [13]. The boxplot, shown in Figure 1a, was also analyzed and displayed a precise median centering. GEO2R generated a volcano plot of every gene demonstrated in Figure 1b and predicted the Log_2_FC and *p*-value for each DEG shown in Table A1. Using |Log_2_FC| ≥ 2 as a differential expression cutoff, 229 DEGs were identified, of which 80 were upregulated in LUAD and 149 were downregulated in LUAD (Table 1).

### 3.2. PPI Analysis of DEGs

**Figure 2:**
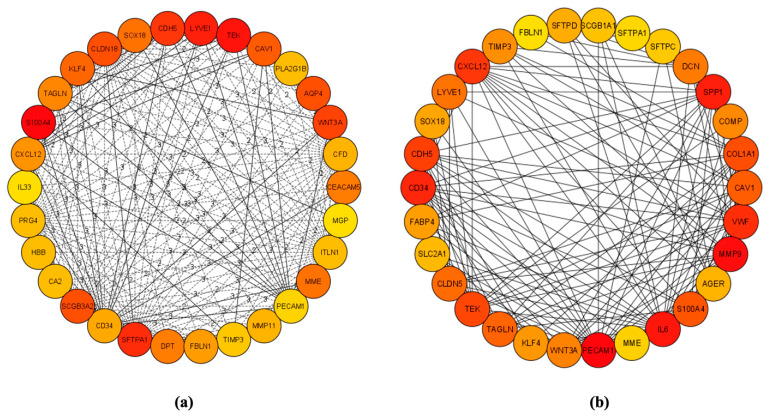
PPI network of hub gene interconnectedness from Cytoscape. All edges (lines) represent protein interactions between specific nodes (proteins). (**a**) DMNC algorithm hub gene predictions; (**b**) MCC algorithm hub gene predictions.

**Figure A1:**
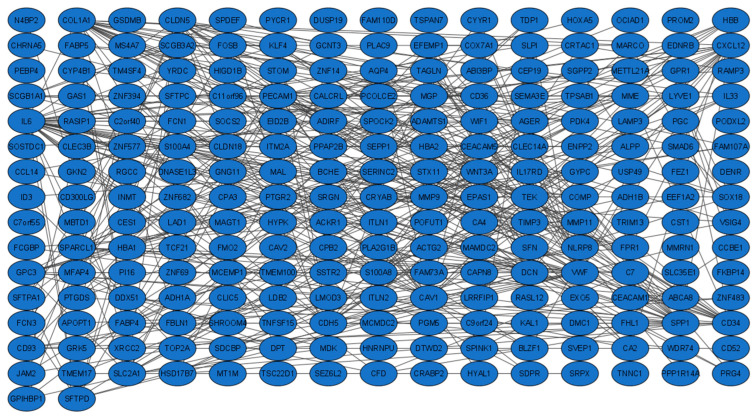
All edges (lines) represent protein interactions between specific nodes (proteins).

Using the STRING database, 229 DEGs were used to construct a PPI network to show interacting proteins, resulting in 227 interacting nodes and 445 edges (Figure A1). The network was exported to Cytoscape for analysis, where cytoHubba was used to screen for hub genes. These are represented by their high connectivity and interactions with other genes in LUAD, as shown by the proteins or nodes with more interactions or edges (Figure 2). CytoHubba’s most robust algorithms, MCC and DMNC, produced two separate PPIs of the hub genes shown in Figure 2. From the two PPIs with some overlapping identifications, 44 total hub genes were identified (Table 2).

### 3.3. Survival Analysis of Hub Genes

**Figure 3:**
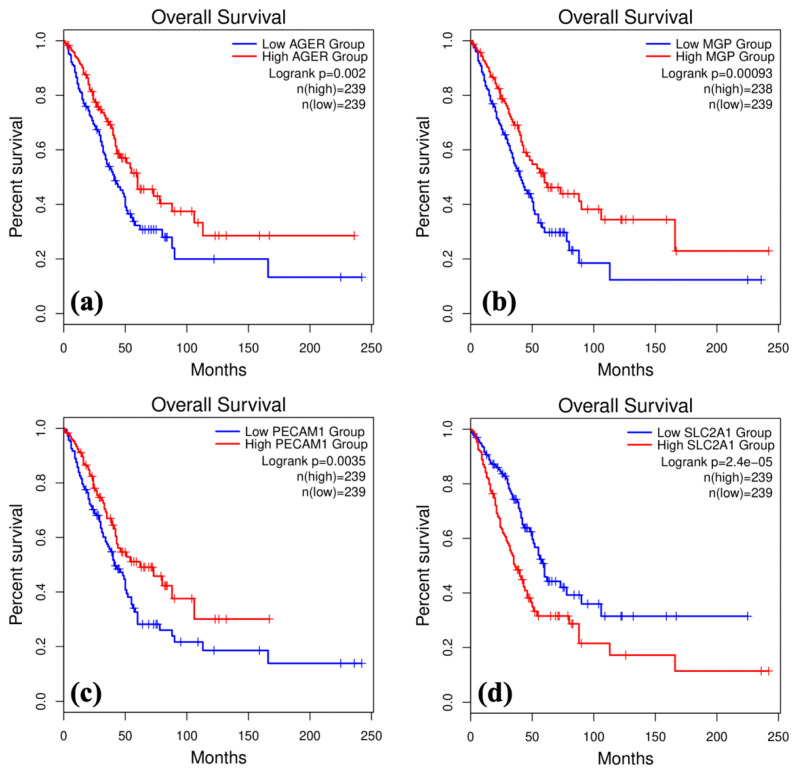
Kaplan–Meier survival analysis of candidate markers. (**a**) *AGER* survival analysis. (**b**) *MGP* survival analysis. (**c**) *PECAM1* survival analysis. (**d**) *SLC2A1* survival analysis.

Using GEPIA-2, the correlation between hub gene expression and LUAD patient survival could be made using 478 patients accessed from TCGA and GTEx. OS analysis indicated that the underexpression of *AGER* (*p* = 0.002), *MGP* (*p* = 0.00093), and *PECAM1* (*p* = 0.0035) correlated with lower survival. Conversely, OS indicated that the overexpression of *SLC2A1* (*p* = 2.4 × 10^−5^) leads to lower survival (Figure 3). From the analysis of all 44 hub genes, *AGER*, *MGP*, *PECAM1*, and *SLC2A1* can be significantly correlated with the prognosis of LUAD, meaning these genes are candidate biomarkers for the early detection of LUAD.

### 3.4. Transcriptomic Verification of Key Genes Using LUAD Samples from TCGA and GTEx

**Figure 4:**
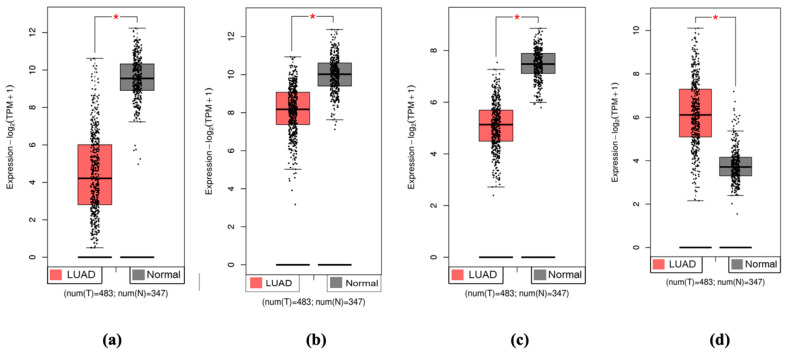
Validation of gene regulation with TCGA and GTEx databases. (**a**) *AGER* downregulation validation. (**b**) *MGP* downregulation validation. (**c**) *PECAM1* downregulation validation. (**d**) *SLC2A1* upregulation validation. * *p* < 0.01.

GEPIA-2 was used to generate differential expression boxplots to validate the candidate biomarker genes for their mRNA differential expression between LUAD and normal lung tissues. GEPIA-2 accessed LUAD and normal lung gene expression data from TCGA and GTEx databases, and the data were used as a test dataset; 483 patients were accessed for tumor tissue gene expression data, and 347 patients were accessed for normal tissue gene expression data. GEPIA-2 produced differential expression boxplots and evaluated for statistical significance to show that *AGER*, *MGP*, and *PECAM1* were downregulated in LUAD compared to normal tissues (Figure 4). GEPIA-2 verified that *SLC2A1* was upregulated in LUAD compared to normal tissues (Figure 4). This verification revealed that *AGER*, *MGP*, and *PECAM1* are significantly downregulated in LUAD and that *SLC2A1* is significantly upregulated in LUAD.

### 3.5. Proteomic Validation of Key Genes Using Immunohistochemistry

**Figure 5:**
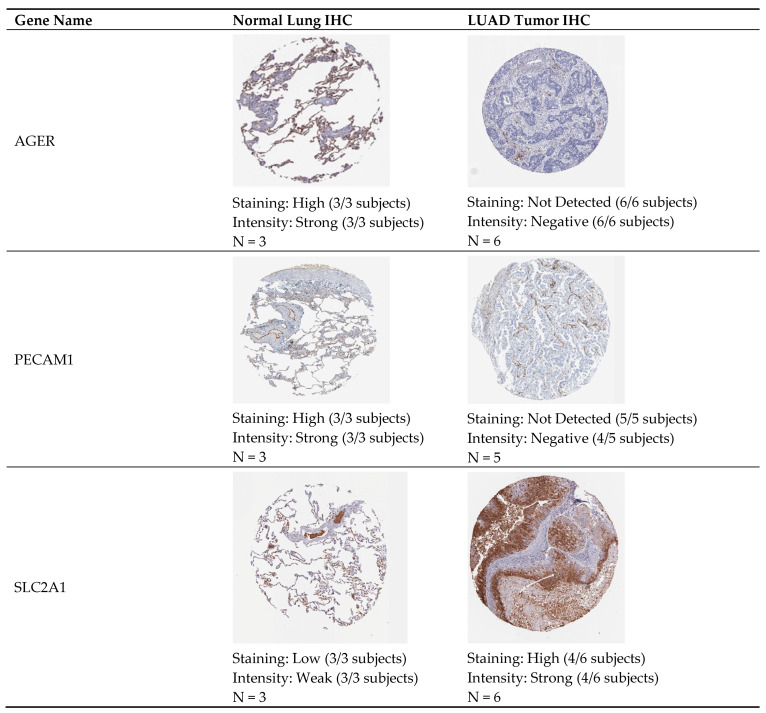
Representative normal lung and tumor cell IHC staining from HPA.

Using the HPA, AGER, MGP, PECAM1, and SLC2A1, normal lung and LUAD tumor IHC staining was accessed. The protein presence was represented by the amount of IHC staining within the normal human lung tissue and LUAD tumor tissue sample. Intensity and level of staining was observed across the biopsy samples. Figure 5 is representative of the overall findings. The IHC showed that AGER and PECAM1 have high intensity and strong staining in all normal lung samples (3/3 subjects), whereas in LUAD tumor tissues, these proteins were absent (0/5–6 showed any positive staining). SLC2A1 had no staining in the normal lung samples (0/3) but had strong expression in the 4/6 LUAD tumor cases. Matching normal lung tissue and LUAD tumor tissue IHC staining for MGP presence was unavailable. According to the extensive bioinformatic analysis, MGP is a highly accurate candidate for its function as a gene biomarker. Overall, AGER, PECAM1, and SLC2A1 were validated for their expression in normal and LUAD tumor IHC samples, showing that the candidate gene biomarkers can function as biologically significant biomarkers to aid in the detection of early-stage LUAD. The robustness of these tissue biomarkers is reinforced by transcriptomic analyses, underscoring their prognostic potential.

## 4. Discussion

This study aimed to identify and validate a novel biomarker signature for early-stage LUAD. We successfully determined a biomarker signature composed of *AGER*, *MGP*, *PECAM1*, and *SLC2A1* that can be utilized to accurately diagnose early-stage LUAD. Using GEO2R, 229 DEGs were identified and networked by the STRING database, and cytoHubba was used to screen 44 hub genes using the MCC and DMNC algorithms. Overall survival (OS) analysis was conducted to verify the correlation of each hub gene’s expression with LUAD prognosis using a TCGA and GTEx test dataset of 478 patient survival data points. *AGER*, *MGP*, *PECAM1*, and *SLC2A1* were the key genes significantly correlated with poorer LUAD prognosis. GEPIA-2 was used to validate the genes’ expression in LUAD by accessing 483 LUAD tumor tissue expression patients and 347 normal lung tissue patients from TCGA and GTEx to act as a test dataset. Lastly, HPA was used to validate the downstream protein expression of each gene using IHC stains of both normal and LUAD tumor tissue. The cumulative approach of identifying genetic biomarkers using GEO, TCGA, GTEx, and HPA allows for the usage of a biologically significant biomarker signature for early-stage LUAD.

There have been several recent studies that identify early diagnostic markers or gene signatures for LUAD that each highlight varying aspects of tumor biology. Li et al. in 2023 used a proteomic profile to propose a panel of secreted proteins that included midkine, WFDC2, and CXCL14 as candidates for early-stage markers for LUAD [9]. Chen et al. performed an integrated analysis of various transcriptomic datasets that elucidated cell cycle-related genes, e.g., *ASPM* and *CCNB2*; these genes were upregulated and associated with a poorer LUAD prognosis [26]. These studies highlight that early-stage LUAD is characterized by diverse molecular changes and provides valuable context for interpreting our biomarker signature.

Superior to other panels, the four-gene signature identified in this study does not overlap with the above candidates, highlighting its novelty [18]. This signature is critically unique because it emphasizes the loss of certain tumor-suppressive factors alongside a known oncogenic driver. *AGER*, *MGP*, and *PECAM1* are significantly downregulated. Downregulating *AGER* and *PECAM1* is highly notable because of their involvement in maintaining normal alveolar cell adhesion and vascular integrity in the lung. The loss of these two genes could potentially facilitate the disorganized growth and angiogenesis that characterizes incipient tumors [20,27]. The upregulation of *SLC2A1* in the signature shows a development in the Warburg effect that can even occur in earlier tumor stages, which is consistent with alternate reports that link *SLC2A1* to aggressive lung cancer behavior [28]. The integration of gene expression, patient survival data, and proteomic validation showed a comprehensive validation of these markers’ clinical relevance. Compared to the existing literature that relied on mRNA data alone, these results demonstrate their significance through rigorous cross-validation.

We list our top four biomarkers as follows: *AGER*, *MGP*, *PECAM1* (downregulated), and *SLC2A1* (upregulated). Advanced Glycosylation End-Product Specific Receptor (AGER) is a transmembrane multi-ligand receptor in the immunoglobulin superfamily. *AGER* is involved in almost every cell type; *AGER* acts as an adhesion molecule in lung epithelium, creating contact between alveolar type I cells and their substrate [20]. This study showed *AGER* is underexpressed in LUAD compared with normal lung tissues; underexpression was correlated with a poorer cancer prognosis (Figure 3). IHC analysis also revealed that AGER had no staining in the LUAD tumor and had high staining in the normal lung, validating the downregulation of the gene in LUAD (Figure 5). *AGER* downregulation has been linked to the loss of cell differentiation and epithelial structure organization simultaneously with oncogenic transformation [20]. Furthermore, existing studies link *AGER* to a poorer prognosis of LUAD, highlighting its biomarker potential [29].

Matrix Gla Protein (MGP) is a protein in the extracellular matrix near vascular tissues. *MGP* is a calcification inhibitor that maintains normal vascular function [30]. This study showed that *MGP* is underexpressed in LUAD compared to normal lung tissues; underexpression was correlated with a poorer cancer prognosis (Figure 3). *MGP* downregulation has been linked to elevated bone morphogenic protein signaling, which is connected to arterial–venous malformations and elevated angiogenesis. *MGP* has also been shown to become upregulated in later-stage cancers to increase tumor stabilization and perfusion [30,31].

Platelet And Endothelial Cell Adhesion Molecule 1 (PECAM1) is a transmembrane protein, part of the immunoglobulin superfamily [32,33]. *PECAM1* connects adjacent endothelial cells and regulates inflammation, leukocyte migration, and vascular responses during sepsis [32]. This study showed *PECAM1* is underexpressed in LUAD compared to normal lung tissues, and the underexpression was linked to poorer cancer prognosis (Figure 3). IHC analysis also revealed that PECAM1 had no staining in the LUAD tumor and had high staining in the normal lung, validating the downregulation of the gene in LUAD (Figure 5). *PECAM1* downregulation has been shown to mediate the secretion of metallopeptidase inhibitor 1, a protein linked to tumor cell proliferation [27].

Solute carrier family 2-facilitated glucose transporter member 1 (SLC2A1) is a glucose transporter highly concentrated in tissue endothelium and epithelium. *SLC2A1* is involved in glucose uptake and allows for aerobic glycolysis [34]. This study showed that *SLC2A1* is overexpressed in LUAD compared to normal lung tissues, and this overexpression was linked to a poorer cancer prognosis (Figure 3). IHC analysis also revealed that SLC2A1 had high staining in the LUAD tumor and had no staining in the normal lung, validating the upregulation of the gene in LUAD (Figure 5). *SLC2A1* overexpression has been linked to transporting more glucose across the cell membrane and allowing for the continuation of aerobic glycolysis and the cell cycle, aiding tumor cell proliferation [34].

Circulating cell-free DNA (cfDNA) is being increasingly utilized in cancer diagnosis, including LUAD. The biomarker signature identified in this study, more specifically AGER and MGP, has unique methylation patterns in tumor versus normal tissues [13,21]. These differences in methylation are detectable in cfDNA and can provide a minimally invasive diagnostic potential to complement imaging techniques such as LDCT screening effectively [35]. Future research may investigate the early-detection performance of these biomarkers in patient plasma cfDNA samples.

Therapeutic strategies targeting the signature could aim to restore the downregulated tumor suppressors or potentially inhibit the upregulated oncogenic pathways in LUAD. Low-dose DNA methyltransferase inhibitors, such as 5-azacytidine, can reverse the gene silencing in lung tumors; analogs of retinoic acid have been shown to halt proliferation mediated by *AGER* and *MGP* [36,37]. The metabolic vulnerability highlighted by the upregulation of *SLC2A1* can be targeted through inhibitors such as WZB117, which impair glucose uptake and could reduce LUAD cell proliferation [38]. The downregulation of *PECAM1* is indicative of an enhanced angiogenesis, which can be mitigated by the multi-kinase inhibitor nintedanib [39]. Targeted small-molecule antagonists of *AGER* show promise in disrupting tumor-promoting *AGER*-mediated signaling [40]. Taken together, these strategies for modulating significant mediators of LUAD prognosis can support the translational potential of this biomarker signature.

The presented biomarker signature shows significant potential as a diagnostic tool in clinical practice. A combined immunohistochemical assay that incorporates AGER, MGP, PECAM1, and SLC2A1 could significantly aid pathology-based distinction between malignant and benign lesions in lung biopsy. Moreover, loss of AGER and PECAM1, coupled with an elevated SLC2A1 in staining, could identify these malignancies effectively in earlier stages of LUAD [29,41].

Additionally, blood-based detection assays that identify altered methylation in AGER and MGP, as well as circulating soluble proteins such as soluble AGER (sRAGE) and MGP, offer minimally invasive options. sRAGE shows potential in allowing for differentiation between LUAD and healthy individuals due to its consistently observed reduction in malignancy [42]. To reduce the prevalence of false positives, these minimally invasive tests could be used as a complement to clinical imaging.

## 5. Conclusions

This study identified and validated a biomarker signature of *AGER*, *MGP*, *PECAM1*, and *SCL2A1* as key genes linked to tumorigenesis and poorer LUAD prognosis. The correlation of these genes to LUAD suggests their clinical usage as biomarkers in detecting early-stage LUAD and are possible targets for therapy. In vivo studies can be used to determine the specific role of these markers in tumor growth, invasion, and response to therapy to further validate these markers.

## Figures and Tables

**Table 1:** Set of upregulated and downregulated genes from GEO2R.

Gene Regulation	DEG Names
Upregulated *	*GCNT3*, *CEACAM5*, *SPP1*, *CST1*, *SPINK1*, *CRABP2*, *MMP11*, *XRCC2*, *COMP*, *SEMA3E*, *FKBP14*, *CHRNA5*, *SHROOM4*, *SFN*, *NLRP8*, *LMOD3*, *LAD1*, *PROM2*, *GSDMB*, *EEF1A2*, *SERINC2*, *TDP1*, *MBTD1*, *ITCH-IT1*, *FCGBP*, *FMC1*, *DTWD2*, *IL17RD*, *HYPK*, *USP49*, *MMP9*, *SEZ6L2*, *APOPT1*, *ZNF682*, *HSD17B7*, *ZNF483*, *ZNF69*, *COL1A1*, *EXO5*, *MAGT1*, *HNRNPU*, *GPR1*, *TM4SF4*, *SLC2A1*, *ZNF14*, *METTL21A*, *ALPP*, *DENR*, *ZNF394*, *BLZF1*, *SPDEF*, *DMC1*, *LRRFIP1*, *MIGA1*, *MDK*, *AOC4P*, *CEACAM1*, *SSTR2*, *TMEM17*, *SLC35E1*, *ZNF577*, *SGPP2*, *CAPN8*, *CEP19*, *DDX51*, *MCMDC*, *PYCR1*, *YRDC*, *WDR74*, *OCIAD1*, *CCBE1*, *N4BP*, *PTGR2*, *DUSP19*, *TOP2A*, *EID2B*, *TRIM13*, *TNFSF1*, *POFUT1*, *PODXL2*
Downregulated *	*ENPP2*, *C11orf96*, *GYPC*, *WNT3A*, *MS4A7*, *CALCRL*, *PLPP3*, *SOSTDC1*, *SFTPD*, *JAM2*, *RASL12*, *TAGLN*, *CD34*, *GRK5*, *STOM*, *ABI3BP*, *CD52*, *BCHE*, *SMAD6*, *HYAL1*, *SLPI*, *TPSAB1*, *FBLN1*, *DPT*, *FCN1*, *KLF4*, *SOCS2*, *PPP1R14A*, *SDCBP*, *PTGDS*, *FPR1*, *VSIG4*, *ADAMTS1*, *CLIC5*, *CYYR1*, *VWF*, *STX11*, *RASIP1*, *CA2*, *LAMP3*, *FEZ1*, *MGP*, *RAMP3*, *PDK4*, *PLA2G1B*, *HOXA5*, *EPAS1*, *PI16*, *S100A4*, *CXCL12*, *GAS1*, *GPC3*, *MAL*, *CLEC14A*, *CES1*, *FABP5*, *MME*, *IL33*, *ANOS1*, *C7*, *ITLN1*, *DNASE1L3*, *SCGB3A2*, *PECAM1*, *FHL1*, *ITM2A*, *EDNRB*, *FAM110D*, *ID3*, *MAMDC2*, *S100A8*, *SVEP1*, *C9orf24*, *SRPX*, *ACTG2*, *AQP4*, *TSC22D1*, *CRYAB*, *MMRN1*, *CD300LG*, *PCOLCE2*, *TSPAN7*, *COX7A1*, *ABCA8*, *CDH5*, *PRG4*, *SOX18*, *CD93*, *PGM5*, *SRGN*, *CYP4B1*, *MARCO*, *ADH1B*, *WIF1*, *SDPR*, *EFEMP1*, *HIGD1B*, *CPB2*, *C2orf40*, *TNNC1*, *CD36*, *RGCC*, *CAV2*, *LDB2*, *TIMP3*, *CLDN5*, *SEPP1*, *SPARCL1*, *CPA3*, *TEK*, *PEBP4*, *CFD*, *FMO2*, *IL6*, *SPOCK2*, *CRTAC1*, *SFTPC*, *AGER*, *DCN*, *LYVE1*, *GPIHBP1*, *MT1M*, *CLDN18*, *PGC*, *TMEM100*, *MFAP4*, *ADIRF*, *ACKR1*, *INMT*, *GNG11*, *FOSB*, *SCGB1A1*, *CCL14*, *TCF21*, *PLAC9*, *GKN2*, *CAV1*, *SFTPA1*, *HBA1*, *ADH1A*, *CLEC3B*, *FABP4*, *HBA2*, *FCN3*, *FAM107A*, *ITLN2*, *MCEMP1*, *CA4*, *HBB*

* Adj. *p* < 0.01.

**Table 2:** Hub genes from MCC and DMNC algorithms based on PPI network.

Hub Gene Name
*VWF*, *DCN*, *FBLN1*, *MME*, *S100A*, *SCGB1A1*, *COMP*, *CXCL12*, *TEK*, *TAGLN*, *AGER*, *SLC2A1*, *IL6*, *SFTPC*, *CLDN5*, *SPP1*, *SFTPD*, *CDH5*, *PECAM1*, *CD34*, *COL1A1*, *CAV1*, *LYVE*, *FABP4*, *WNT3A*, *TIMP3*, *KLF4*, *MMP9*, *SFTPA1*, *SOX18*, *MGP*, *DPT*, *ITLN1*, *IL33*, *SCGB3A2*, *CFD*, *CA2*, *PRG4*, *MMP11*, *CEACAM5*, *AQP4*, *PLA2G1B*, *HBB*, *CLDN*

## Data Availability

The datasets analyzed during the current study are available in the Gene Expression Omnibus under accession number GSE32863 (https://www.ncbi.nlm.nih.gov/geo/query/acc.cgi?acc=GSE32863, accessed on 22 September 2024) and are published by Selamat et al. (DOI: 10.1101/gr.132662.111).

## Abbreviations

The following abbreviations are used in this manuscript:

NSCLC	Non-Small-Cell Lung Cancer
LUAD	Lung Adenocarcinoma
AAH	Atypical Adenomatous Hyperplasia
AIS	Adenocarcinoma In Situ
LDCT	Low-Dose CT
GEO	Gene Expression Omnibus
DEG	Differentially Expressed Gene
Log_2_FC	Log_2_ Fold Change
STRING	Search Tool for the Retrieval of Interacting Genes/Proteins
PPI	Protein–Protein Interaction
MCC	Maximal Clique Centrality
DMNC	Density of Maximum Neighborhood Component
GEPIA-2	Gene Expression Profiling Interactive Analysis-2
OS	Overall Survival
TCGA	The Cancer Genome Atlas
GTEx	Genotype-Tissue Expression Database
IHC	Immunohistochemistry
HPA	Human Protein Atlas
AGER	Advanced Glycosylation End-Product Specific Receptor
MGP	Matrix Gla Protein
PECAM1	Platelet and Endothelial Cell Adhesion Molecule 1
SLC2A1	Solute Carrier Family 2-Facilitated Glucose Transporter Member 1
cfDNA	Circulating Cell-Free DNA
sRAGE	Soluble Receptor for Advanced Glycation End-Products

## Appendix A

**Table A1:** GEO2R annotation of all significant DEGs.

Gene Symbol	Gene Title	*p*-Value	Log_2_FC
GCNT3	Glucosaminyl (N-acetyl) transferase 3, mucin type	6.25 × 10^−10^	3.47
CEACAM5	Carcinoembryonic antigen-related cell adhesion molecule 5	2.55 × 10^−7^	3.45
SPP1	Secreted phosphoprotein 1	2.61 × 10^−8^	3.38
CST1	Cystatin SN	4.11 × 10^−6^	2.9
SPINK1	Serine peptidase inhibitor, Kazal type 1	9.49 × 10^−5^	2.85
CRABP2	Cellular retinoic acid-binding protein 2	7.23 × 10^−9^	2.75
MMP11	Matrix metallopeptidase 11	1.30 × 10^−7^	2.72
XRCC2	X-ray repair cross-complementing 2	1.65 × 10^−7^	2.62
COMP	Cartilage oligomeric matrix protein	3.81 × 10^−6^	2.59
SEMA3E	Semaphorin 3E	1.83 × 10^−7^	2.57
FKBP14	FK506-binding protein 14	1.88 × 10^−7^	2.54
CHRNA5	Cholinergic receptor nicotinic alpha 5 subunit	7.94 × 10^−8^	2.51
SHROOM4	Shroom family member 4	7.24 × 10^−8^	2.44
SFN	Stratifin	1.64 × 10^−11^	2.43
NLRP8	NLR family pyrin domain-containing 8	1.41 × 10^−7^	2.43
LMOD3	Leiomodin 3	6.95 × 10^−7^	2.43
LAD1	Ladinin 1	2.39 × 10^−13^	2.42
PROM2	Prominin 2	6.75 × 10^−12^	2.42
GSDMB	Gasdermin B	9.54 × 10^−12^	2.42
EEF1A2	Eukaryotic translation elongation factor 1 alpha 2	7.99 × 10^−5^	2.42
SERINC2	Serine incorporator 2	5.37 × 10^−10^	2.4
TDP1	Tyrosyl-DNA phosphodiesterase 1	2.74 × 10^−7^	2.39
MBTD1	mbt domain-containing 1	2.95 × 10^−7^	2.39
ITCH-IT1	ITCH intronic transcript 1	2.07 × 10^−6^	2.34
FCGBP	Fc fragment of IgG-binding protein	5.37 × 10^−7^	2.33
FMC1	Formation of mitochondrial complex V assembly factor 1 homolog	5.55 × 10^−7^	2.33
DTWD2	DTW domain-containing 2	5.68 × 10^−7^	2.33
IL17RD	Interleukin 17 receptor D	1.28 × 10^−6^	2.31
HYPK	Huntingtin-interacting protein K	8.37 × 10^−9^	2.3
USP49	Ubiquitin-specific peptidase 49	3.78 × 10^−7^	2.3
MMP9	Matrix metallopeptidase 9	3.94 × 10^−6^	2.3
SEZ6L2	Seizure-related 6 homolog-like 2	1.18 × 10^−8^	2.29
APOPT1	Apoptogenic 1, mitochondrial	1.38 × 10^−7^	2.27
ZNF682	Zinc finger protein 682	2.15 × 10^−6^	2.27
HSD17B7	Hydroxysteroid 17-beta dehydrogenase 7	1.54 × 10^−7^	2.26
ZNF483	Zinc finger protein 483	9.67 × 10^−7^	2.26
ZNF69	Zinc finger protein 69	3.20 × 10^−8^	2.24
COL1A1	Collagen type I alpha 1 chain	8.75 × 10^−7^	2.24
EXO5	Exonuclease 5	1.05 × 10^−6^	2.24
MAGT1	Magnesium transporter 1	1.23 × 10^−7^	2.23
HNRNPU	Heterogeneous nuclear ribonucleoprotein U	2.04 × 10^−6^	2.23
GPR1	G protein-coupled receptor 1	6.78 × 10^−7^	2.22
TM4SF4	Transmembrane 4 L six family member 4	1.28 × 10^−3^	2.21
SLC2A1	Solute carrier family 2 member 1	2.91 × 10^−8^	2.2
ZNF14	Zinc finger protein 14	7.02 × 10^−8^	2.2
METTL21A	Methyltransferase-like 21A	1.17 × 10^−7^	2.2
ALPP	Alkaline phosphatase, placental	1.65 × 10^−7^	2.2
DENR	Density regulated re-initiation and release factor	8.76 × 10^−7^	2.2
ZNF394	Zinc finger protein 394	2.44 × 10^−7^	2.19
BLZF1	Basic leucine zipper nuclear factor 1	3.08 × 10^−7^	2.19
SPDEF	SAM pointed domain-containing ETS transcription factor	4.41 × 10^−8^	2.18
DMC1	DNA meiotic recombinase 1	2.22 × 10^−7^	2.16
LRRFIP1	LRR-binding FLII-interacting protein 1	2.72 × 10^−7^	2.16
MIGA1	Mitoguardin 1	4.61 × 10^−6^	2.14
MDK	Midkine (neurite growth-promoting factor 2)	3.50 × 10^−11^	2.13
AOC4P	Amine oxidase, copper-containing 4, pseudogene	8.05 × 10^−7^	2.13
CEACAM1	Carcinoembryonic antigen-related cell adhesion molecule 1	9.58 × 10^−11^	2.12
SSTR2	Somatostatin receptor 2	2.99 × 10^−6^	2.12
TMEM17	Transmembrane protein 17	1.99 × 10^−7^	2.11
SLC35E1	Solute carrier family 35 member E1	3.62 × 10^−7^	2.11
ZNF577	Zinc finger protein 577	2.81 × 10^−7^	2.11
SGPP2	Sphingosine-1-phosphate phosphatase 2	8.66 × 10^−15^	2.1
CAPN8	Calpain 8	6.39 × 10^−7^	2.1
CEP19	Centrosomal protein 19	6.05 × 10^−8^	2.09
DDX51	DEAD-box helicase 51	5.58 × 10^−8^	2.07
MCMDC2	Minichromosome maintenance domain-containing 2	3.28 × 10^−7^	2.07
PYCR1	Pyrroline-5-carboxylate reductase 1	3.82 × 10^−15^	2.06
YRDC	yrdC N6-threonylcarbamoyltransferase domain-containing	9.04 × 10^−7^	2.06
WDR74	WD repeat domain 74	3.59 × 10^−6^	2.06
OCIAD1	OCIA domain-containing 1	9.42 × 10^−8^	2.05
CCBE1	Collagen- and calcium-binding EGF domains 1	4.29 × 10^−6^	2.05
N4BP2	NEDD4-binding protein 2	1.46 × 10^−6^	2.04
PTGR2	Prostaglandin reductase 2	1.18 × 10^−5^	2.04
DUSP19	Dual-specificity phosphatase 19	1.19 × 10^−7^	2.03
TOP2A	Topoisomerase (DNA) II alpha	3.55 × 10^−9^	2.02
EID2B	EP300-interacting inhibitor of differentiation 2B	1.33 × 10^−7^	2.01
TRIM13	Tripartite motif-containing 13	1.96 × 10^−7^	2.01
TNFSF15	Tumor necrosis factor superfamily member 15	8.00 × 10^−6^	2.01
POFUT1	Protein O-fucosyltransferase 1	9.23 × 10^−8^	2
PODXL2	Podocalyxin-like 2	3.46 × 10^−6^	2
ENPP2	Ectonucleotide pyrophosphatase/phosphodiesterase 2	2.81 × 10^−15^	−2
C11orf96	Chromosome 11 open reading frame 96	1.56 × 10^−12^	−2
GYPC	Glycophorin C (Gerbich blood group)	2.70 × 10^−19^	−2
WNT3A	Wnt family member 3A	2.40 × 10^−15^	−2
MS4A7	Membrane spanning 4-domains A7	1.07 × 10^−12^	−2
CALCRL	Calcitonin receptor-like receptor	1.11 × 10^−10^	−2
PLPP3	Phospholipid phosphatase 3	3.14 × 10^−10^	−2
SOSTDC1	Sclerostin domain-containing 1	3.51 × 10^−12^	−2
SFTPD	Surfactant protein D	1.97 × 10^−10^	−2
JAM2	Junctional adhesion molecule 2	3.65 × 10^−9^	−2
RASL12	RAS-like family 12	4.78 × 10^−5^	−2
TAGLN	Transgelin	8.07 × 10^−9^	−2.1
CD34	CD34 molecule	1.78 × 10^−7^	−2.1
GRK5	G protein-coupled receptor kinase 5	3.86 × 10^−7^	−2.1
STOM	Stomatin	1.07 × 10^−10^	−2.1
ABI3BP	ABI family member 3-binding protein	3.73 × 10^−11^	−2.1
CD52	CD52 molecule	4.08 × 10^−7^	−2.1
BCHE	Butyrylcholinesterase	4.72 × 10^−11^	−2.1
SMAD6	SMAD family member 6	1.69 × 10^−7^	−2.1
HYAL1	Hyaluronoglucosaminidase 1	2.47 × 10^−9^	−2.1
SLPI	Secretory leukocyte peptidase inhibitor	9.70 × 10^−9^	−2.1
TPSAB1	Tryptase alpha/beta 1	3.32 × 10^−8^	−2.1
FBLN1	Fibulin 1	1.06 × 10^−7^	−2.1
DPT	Dermatopontin	2.72 × 10^−11^	−2.1
FCN1	Ficolin 1	3.36 × 10^−11^	−2.1
KLF4	Kruppel-like factor 4	4.62 × 10^−11^	−2.1
SOCS2	Suppressor of cytokine signaling 2	1.39 × 10^−9^	−2.1
PPP1R14A	Protein phosphatase 1 regulatory inhibitor subunit 14A	8.61 × 10^−13^	−2.1
SDCBP	Syndecan-binding protein	7.32 × 10^−9^	−2.1
PTGDS	Prostaglandin D2 synthase	3.03 × 10^−12^	−2.1
FPR1	Formyl peptide receptor 1	5.96 × 10^−7^	−2.2
VSIG4	V-set and immunoglobulin domain-containing 4	5.97 × 10^−5^	−2.2
ADAMTS1	ADAM metallopeptidase with thrombospondin type 1 motif 1	3.52 × 10^−15^	−2.2
CLIC5	Chloride intracellular channel 5	2.13 × 10^−12^	−2.2
CYYR1	Cysteine and tyrosine rich 1	1.30 × 10^−8^	−2.2
VWF	von Willebrand factor	6.63 × 10^−7^	−2.2
STX11	Syntaxin 11	6.98 × 10^−9^	−2.2
RASIP1	Ras-interacting protein 1	4.49 × 10^−7^	−2.2
CA2	Carbonic anhydrase 2	3.23 × 10^−12^	−2.2
LAMP3	Lysosomal-associated membrane protein 3	1.65 × 10^−11^	−2.2
FEZ1	Fasciculation and elongation protein zeta 1	5.42 × 10^−11^	−2.2
MGP	Matrix Gla protein	1.87 × 10^−10^	−2.2
RAMP3	Receptor (G protein-coupled) activity-modifying protein 3	2.51 × 10^−8^	−2.3
PDK4	Pyruvate dehydrogenase kinase 4	4.03 × 10^−5^	−2.3
PLA2G1B	Phospholipase A2 group IB	2.12 × 10^−12^	−2.3
HOXA5	Homeobox A5	6.17 × 10^−7^	−2.3
EPAS1	Endothelial PAS domain protein 1	2.81 × 10^−14^	−2.3
PI16	Peptidase inhibitor 16	6.75 × 10^−12^	−2.3
S100A4	S100 calcium-binding protein A4	1.70 × 10^−11^	−2.3
CXCL12	C-X-C motif chemokine ligand 12	5.52 × 10^−11^	−2.3
GAS1	Growth arrest-specific 1	3.34 × 10^−7^	−2.3
GPC3	Glypican 3	2.41 × 10^−17^	−2.3
MAL	mal, T-cell differentiation protein	1.85 × 10^−12^	−2.3
CLEC14A	C-type lectin domain family 14 member A	2.38 × 10^−11^	−2.3
CES1	Carboxylesterase 1	3.23 × 10^−10^	−2.3
FABP5	Fatty acid-binding protein 5	9.86 × 10^−10^	−2.3
MME	Membrane metallo-endopeptidase	2.38 × 10^−8^	−2.3
IL33	Interleukin 33	5.05 × 10^−4^	−2.3
ANOS1	Anosmin 1	5.92 × 10^−15^	−2.3
C7	Complement component 7	2.52 × 10^−12^	−2.3
ITLN1	Intelectin 1	4.14 × 10^−10^	−2.3
DNASE1L3	Deoxyribonuclease 1-like 3	6.60 × 10^−14^	−2.4
SCGB3A2	Secretoglobin family 3A member 2	1.08 × 10^−10^	−2.4
PECAM1	Platelet and endothelial cell adhesion molecule 1	1.42 × 10^−4^	−2.4
FHL1	Four-and-a-half LIM domains 1	2.20 × 10^−4^	−2.4
ITM2A	Integral membrane protein 2A	8.81 × 10^−16^	−2.4
EDNRB	Endothelin receptor type B	1.64 × 10^−11^	−2.4
FAM110D	Family with sequence similarity 110 member D	6.27 × 10^−10^	−2.4
ID3	Inhibitor of DNA-binding 3, HLH protein	6.42 × 10^−13^	−2.4
MAMDC2	MAM domain-containing 2	1.10 × 10^−4^	−2.4
S100A8	S100 calcium-binding protein A8	2.93 × 10^−10^	−2.4
SVEP1	Sushi, von Willebrand factor type A, EGF, and pentraxin domain-containing 1	2.13 × 10^−8^	−2.4
C9orf24	Chromosome 9 open reading frame 24	1.42 × 10^−6^	−2.4
SRPX	Sushi repeat-containing protein, X-linked	8.31 × 10^−17^	−2.5
ACTG2	Actin, gamma 2, smooth muscle, enteric	2.37 × 10^−12^	−2.5
AQP4	Aquaporin 4	5.42 × 10^−11^	−2.5
TSC22D1	TSC22 domain family member 1	1.33 × 10^−10^	−2.5
CRYAB	Crystallin alpha B	3.23 × 10^−12^	−2.5
MMRN1	Multimerin 1	8.74 × 10^−17^	−2.5
CD300LG	CD300 molecule-like family member g	7.31 × 10^−19^	−2.5
PCOLCE2	Procollagen C-endopeptidase enhancer 2	2.97 × 10^−17^	−2.5
TSPAN7	Tetraspanin 7	1.53 × 10^−14^	−2.5
COX7A1	Cytochrome c oxidase subunit 7A1	1.68 × 10^−13^	−2.5
ABCA8	ATP-binding cassette subfamily A member 8	3.43 × 10^−10^	−2.5
CDH5	Cadherin 5	2.81 × 10^−14^	−2.5
PRG4	Proteoglycan 4	2.90 × 10^−12^	−2.6
SOX18	SRY-box 18	2.85 × 10^−10^	−2.6
CD93	CD93 molecule	1.95 × 10^−8^	−2.6
PGM5	Phosphoglucomutase 5	1.33 × 10^−7^	−2.6
SRGN	Serglycin	2.14 × 10^−10^	−2.6
CYP4B1	Cytochrome P450 family 4 subfamily B member 1	5.78 × 10^−18^	−2.6
MARCO	Macrophage receptor with collagenous structure	4.90 × 10^−9^	−2.6
ADH1B	Alcohol dehydrogenase 1B (class I), beta polypeptide	5.60 × 10^−15^	−2.6
WIF1	WNT inhibitory factor 1	1.29 × 10^−8^	−2.6
SDPR	Serum deprivation response	3.88 × 10^−16^	−2.6
EFEMP1	EGF-containing fibulin-like extracellular matrix protein 1	5.36 × 10^−14^	−2.6
HIGD1B	HIG1 hypoxia-inducible domain family member 1B	2.13 × 10^−13^	−2.6
CPB2	Carboxypeptidase B2	2.12 × 10^−10^	−2.6
C2orf40	Chromosome 2 open reading frame 40	8.89 × 10^−15^	−2.6
TNNC1	Troponin C1, slow skeletal and cardiac type	1.59 × 10^−14^	−2.7
CD36	CD36 molecule	1.85 × 10^−12^	−2.7
RGCC	Regulator of cell cycle	5.90 × 10^−12^	−2.7
CAV2	Caveolin 2	2.43 × 10^−11^	−2.7
LDB2	LIM domain-binding 2	2.12 × 10^−12^	−2.7
TIMP3	TIMP metallopeptidase inhibitor 3	1.06 × 10^−12^	−2.7
CLDN5	Claudin 5	8.58 × 10^−12^	−2.7
SEPP1	Selenoprotein P, plasma, 1	6.48 × 10^−18^	−2.7
SPARCL1	SPARC-like 1	2.92 × 10^−14^	−2.7
CPA3	Carboxypeptidase A3	2.36 × 10^−11^	−2.8
TEK	TEK receptor tyrosine kinase	4.42 × 10^−15^	−2.8
PEBP4	Phosphatidylethanolamine-binding protein 4	4.29 × 10^−11^	−2.8
CFD	Complement factor D	1.26 × 10^−16^	−2.9
FMO2	Flavin-containing monooxygenase 2	1.74 × 10^−15^	−2.9
IL6	Interleukin 6	1.28 × 10^−11^	−2.9
SPOCK2	Sparc/osteonectin-, cwcv-, and kazal-like domains proteoglycan (testican) 2	1.89 × 10^−12^	−2.9
CRTAC1	Cartilage acidic protein 1	4.74 × 10^−5^	−2.9
SFTPC	Surfactant protein C	5.93 × 10^−17^	−2.9
AGER	Advanced glycosylation end-product-specific receptor	9.41 × 10^−18^	−3
DCN	Decorin	2.18 × 10^−15^	−3
LYVE1	Lymphatic vessel endothelial hyaluronan receptor 1	2.02 × 10^−9^	−3
GPIHBP1	Glycosylphosphatidylinositol-anchored high-density lipoprotein-binding protein 1	7.41 × 10^−7^	−3
MT1M	Metallothionein 1M	5.58 × 10^−12^	−3
CLDN18	Claudin 18	1.46 × 10^−5^	−3
PGC	Progastricsin	1.85 × 10^−12^	−3
TMEM100	Transmembrane protein 100	8.56 × 10^−14^	−3
MFAP4	Microfibrillar-associated protein 4	2.52 × 10^−12^	−3.1
ADIRF	Adipogenesis regulatory factor	5.46 × 10^−12^	−3.1
ACKR1	Atypical chemokine receptor 1 (Duffy blood group)	5.58 × 10^−12^	−3.1
INMT	Indolethylamine N-methyltransferase	2.78 × 10^−14^	−3.1
GNG11	G protein subunit gamma 11	3.44 × 10^−6^	−3.2
FOSB	FosB proto-oncogene, AP-1 transcription factor subunit	1.51 × 10^−17^	−3.2
SCGB1A1	Secretoglobin family 1A member 1	3.02 × 10^−17^	−3.2
CCL14	C-C motif chemokine ligand 14	1.79 × 10^−6^	−3.2
TCF21	Transcription factor 21	1.65 × 10^−8^	−3.4
PLAC9	Placenta-specific 9	6.10 × 10^−14^	−3.5
GKN2	Gastrokine 2	3.61 × 10^−11^	−3.7
CAV1	Caveolin 1	1.85 × 10^−10^	−3.7
SFTPA1	Surfactant protein A1	5.78 × 10^−18^	−3.7
HBA1	Hemoglobin subunit alpha 1	8.81 × 10^−16^	−3.7
ADH1A	Alcohol dehydrogenase 1A (class I), alpha polypeptide	3.11 × 10^−21^	−3.7
CLEC3B	C-type lectin domain family 3 member B	2.41 × 10^−17^	−3.7
FABP4	Fatty acid-binding protein 4	9.37 × 10^−12^	−3.8
HBA2	Hemoglobin subunit alpha 2	5.69 × 10^−14^	−3.8
FCN3	Ficolin 3	3.10 × 10^−19^	−3.8
FAM107A	Family with sequence similarity 107 member A	3.11 × 10^−21^	−4.1
ITLN2	Intelectin 2	4.32 × 10^−16^	−4.1
MCEMP1	Mast cell-expressed membrane protein 1	1.75 × 10^−13^	−4.2
CA4	Carbonic anhydrase 4	2.41 × 10^−10^	−4.2
HBB	Hemoglobin subunit beta	5.78 × 10^−18^	−4.4

## References

[1] Siegel R.L., Giaquinto A.N., Jemal A. (2024). Cancer statistics, 2024. CA Cancer J. Clin..

[2] Li C., Lei S., Ding L., Xu Y., Wu X., Wang H., Zhang Z., Gao T., Zhang Y., Li L. (2023). Global burden and trends of lung cancer incidence and mortality. Chin. Med. J..

[3] Freitas C., Sousa C., Machado F., Serino M., Santos V., Cruz-Martins N., Teixeira A., Cunha A., Pereira T., Oliveira H.P. (2021). The Role of Liquid Biopsy in Early Diagnosis of Lung Cancer. Front. Oncol..

[4] Dubin S., Griffin D. (2020). Lung Cancer in Non-Smokers. Mo. Med..

[5] Kontomanolis E.N., Koutras A., Syllaios A., Schizas D., Mastoraki A., Garmpis N., Diakosavvas M., Angelou K., Tsatsaris G., Pagkalos A. (2020). Role of Oncogenes and Tumor-suppressor Genes in Carcinogenesis: A Review. Anticancer Res..

[6] Travis W.D., Brambilla E., Nicholson A.G., Yatabe Y., Austin J.H.M., Beasley M.B., Chirieac L.R., Dacic S., Duhig E., Flieder D.B. (2015). The 2015 World Health Organization Classification of Lung Tumors: Impact of Genetic, Clinical and Radiologic Advances Since the 2004 Classification. J. Thorac. Oncol..

[7] Liang P., Peng M., Tao J., Wang B., Wei J., Lin L., Cheng B., Xiong S., Li J., Li C. (2024). Development of a genome atlas for discriminating benign, preinvasive, and invasive lung nodules. MedComm (2020).

[8] Gong J., Yu D. (2024). Mapping the immune terrain in lung adenocarcinoma progression: Tfh-like cells in tertiary lymphoid structures. Cell Oncol..

[9] Li J., Li J., Hao H., Lu F., Wang J., Ma M., Jia B., Zhuo M., Wang J., Chi Y. (2023). Secreted proteins MDK, WFDC2, and CXCL14 as candidate biomarkers for early diagnosis of lung adenocarcinoma. BMC Cancer.

[10] Boutsikou E., Hardavella G., Fili E., Bakiri A., Gaitanakis S., Kote A., Samitas K., Gkiozos I. (2024). The Role of Biomarkers in Lung Cancer Screening. Cancers.

[11] Huang Y., Ma S., Xu J.Y., Qian K., Wang Y., Zhang Y., Tan M., Xiao T. (2024). Prognostic biomarker discovery based on proteome landscape of Chinese lung adenocarcinoma. Clin. Proteom..

[12] Mo L., Wei B., Liang R., Yang Z., Xie S., Wu S., You Y. (2020). Exploring potential biomarkers for lung adenocarcinoma using LC-MS/MS metabolomics. J. Int. Med. Res..

[13] Selamat S.A., Chung B.S., Girard L., Zhang W., Zhang Y., Campan M., Siegmund K.D., Koss M.N., Hagen J.A., Lam W.L. (2012). Genome-scale analysis of DNA methylation in lung adenocarcinoma and integration with mRNA expression. Genome Res..

[14] Shen Y., Dong S., Liu J., Zhang L., Zhang J., Zhou H., Dong W. (2020). Identification of Potential Biomarkers for Thyroid Cancer Using Bioinformatics Strategy: A Study Based on GEO Datasets. Biomed. Res. Int..

[15] Skanland S.S., Cremaschi A., Bendiksen H., Hermansen J.U., Thimiri Govinda Raj D.B., Munthe L.A., Tjonnfjord G.E., Tasken K. (2020). An in vitro assay for biomarker discovery and dose prediction applied to ibrutinib plus venetoclax treatment of CLL. Leukemia.

[16] Zhang Y., Zheng Y., Fu Y., Wang C. (2019). Identification of biomarkers, pathways and potential therapeutic agents for white adipocyte insulin resistance using bioinformatics analysis. Adipocyte.

[17] Ding L., Getz G., Wheeler D.A., Mardis E.R., McLellan M.D., Cibulskis K., Sougnez C., Greulich H., Muzny D.M., Morgan M.B. (2008). Somatic mutations affect key pathways in lung adenocarcinoma. Nature.

[18] Hamad W., Grigore B., Walford H., Peters J., Alexandris P., Bonfield S., Standen L., Boscott R., Behiyat D., Kuhn I. (2025). Biomarkers Suitable for Early Detection of Intrathoracic Cancers in Primary Care: A Systematic Review. Cancer Epidemiol. Biomarkers Prev..

[19] Cao W., Tang Q., Zeng J., Jin X., Zu L., Xu S. (2023). A Review of Biomarkers and Their Clinical Impact in Resected Early-Stage Non-Small-Cell Lung Cancer. Cancers.

[20] Marinakis E., Bagkos G., Piperi C., Roussou P., Diamanti-Kandarakis E. (2014). Critical role of RAGE in lung physiology and tumorigenesis: A potential target of therapeutic intervention?. Clin. Chem. Lab. Med..

[21] Caiado H., Cancela M.L., Conceicao N. (2023). Assessment of MGP gene expression in cancer and contribution to prognosis. Biochimie.

[22] Cahill C.M., Sarang S.S., Bakshi R., Xia N., Lahiri D.K., Rogers J.T. (2024). Neuroprotective Strategies and Cell-Based Biomarkers for Manganese-Induced Toxicity in Human Neuroblastoma (SH-SY5Y) Cells. Biomolecules.

[23] Szklarczyk D., Gable A.L., Lyon D., Junge A., Wyder S., Huerta-Cepas J., Simonovic M., Doncheva N.T., Morris J.H., Bork P. (2019). STRING v11: Protein-protein association networks with increased coverage, supporting functional discovery in genome-wide experimental datasets. Nucleic Acids Res..

[24] Tang Z., Li C., Kang B., Gao G., Li C., Zhang Z. (2017). GEPIA: A web server for cancer and normal gene expression profiling and interactive analyses. Nucleic Acids Res..

[25] Thul P.J., Lindskog C. (2018). The human protein atlas: A spatial map of the human proteome. Protein Sci..

[26] Chen Y., Jin L., Jiang Z., Liu S., Feng W. (2021). Identifying and Validating Potential Biomarkers of Early Stage Lung Adenocarcinoma Diagnosis and Prognosis. Front. Oncol..

[27] Abraham V., Cao G., Parambath A., Lawal F., Handumrongkul C., Debs R., DeLisser H.M. (2018). Involvement of TIMP-1 in PECAM-1-mediated tumor dissemination. Int. J. Oncol..

[28] Mathew M., Nguyen N.T., Bhutia Y.D., Sivaprakasam S., Ganapathy V. (2024). Metabolic Signature of Warburg Effect in Cancer: An Effective and Obligatory Interplay between Nutrient Transporters and Catabolic/Anabolic Pathways to Promote Tumor Growth. Cancers.

[29] Wang Q., Zhu W., Xiao G., Ding M., Chang J., Liao H. (2020). Effect of AGER on the biological behavior of non-small cell lung cancer H1299 cells. Mol. Med. Rep..

[30] Sharma B., Albig A.R. (2013). Matrix Gla protein reinforces angiogenic resolution. Microvasc Res..

[31] Jaminon A.M.G., Dai L., Qureshi A.R., Evenepoel P., Ripsweden J., Soderberg M., Witasp A., Olauson H., Schurgers L.J., Stenvinkel P. (2020). Matrix Gla protein is an independent predictor of both intimal and medial vascular calcification in chronic kidney disease. Sci. Rep..

[32] Villar J., Zhang H., Slutsky A.S. (2019). Lung Repair and Regeneration in ARDS: Role of PECAM1 and Wnt Signaling. Chest.

[33] Woodfin A., Voisin M.B., Nourshargh S. (2007). PECAM-1: A multi-functional molecule in inflammation and vascular biology. Arterioscler. Thromb. Vasc. Biol..

[34] Wang Y., Shi S., Ding Y., Wang Z., Liu S., Yang J., Xu T. (2017). Metabolic reprogramming induced by inhibition of SLC2A1 suppresses tumor progression in lung adenocarcinoma. Int. J. Clin. Exp. Pathol..

[35] Jin Y., Lu R., Liu F., Jiang G., Wang R., Zheng M. (2024). DNA methylation analysis in plasma for early diagnosis in lung adenocarcinoma. Medicine.

[36] Mahesh S., Saxena A., Qiu X., Perez-Soler R., Zou Y. (2010). Intratracheally administered 5-azacytidine is effective against orthotopic human lung cancer xenograft models and devoid of important systemic toxicity. Clin. Lung Cancer.

[37] Zito G., Naselli F., Saieva L., Raimondo S., Calabrese G., Guzzardo C., Forte S., Rolfo C., Parenti R., Alessandro R. (2017). Retinoic Acid affects Lung Adenocarcinoma growth by inducing differentiation via GATA6 activation and EGFR and Wnt inhibition. Sci. Rep..

[38] Liu Y., Cao Y., Zhang W., Bergmeier S., Qian Y., Akbar H., Colvin R., Ding J., Tong L., Wu S. (2012). A small-molecule inhibitor of glucose transporter 1 downregulates glycolysis, induces cell-cycle arrest, and inhibits cancer cell growth in vitro and in vivo. Mol. Cancer Ther..

[39] Reck M., Kaiser R., Mellemgaard A., Douillard J.Y., Orlov S., Krzakowski M., von Pawel J., Gottfried M., Bondarenko I., Liao M. (2014). Docetaxel plus nintedanib versus docetaxel plus placebo in patients with previously treated non-small-cell lung cancer (LUME-Lung 1): A phase 3, double-blind, randomised controlled trial. Lancet Oncol..

[40] Manigrasso M.B., Pan J., Rai V., Zhang J., Reverdatto S., Quadri N., DeVita R.J., Ramasamy R., Shekhtman A., Schmidt A.M. (2016). Small Molecule Inhibition of Ligand-Stimulated RAGE-DIAPH1 Signal Transduction. Sci. Rep..

[41] Tan Z., Yang C., Zhang X., Zheng P., Shen W. (2017). Expression of glucose transporter 1 and prognosis in non-small cell lung cancer: A pooled analysis of 1665 patients. Oncotarget.

[42] Jing R., Cui M., Wang J., Wang H. (2010). Receptor for advanced glycation end products (RAGE) soluble form (sRAGE): A new biomarker for lung cancer. Neoplasma.

